# Machine learning–based insights into circulating autoantibody dynamics and treatment outcomes in patients with NSCLC receiving immune checkpoint inhibitors

**DOI:** 10.3389/fimmu.2025.1666030

**Published:** 2025-10-03

**Authors:** Feifei Wei, Hiroyuki Takeda, Koichi Azuma, Yoshiro Nakahara, Yuka Igarashi, Kenta Murotani, Haruhiro Saito, Shuji Murakami, Tetsuro Kondo, Taku Kouro, Hidetomo Himuro, Kayoko Tsuji, Mitsuru Komahashi, Tatsuya Sawasaki, Tetsuro Sasada

**Affiliations:** ^1^ Division of Cancer Immunotherapy, Kanagawa Cancer Center Research Institute, Yokohama, Kanagawa, Japan; ^2^ Cancer Vaccine and Immunotherapy Center, Kanagawa Cancer Center, Yokohama, Kanagawa, Japan; ^3^ Division of Proteo-Drug-Discovery Sciences, Proteo-Science Center, PIAS, Ehime University, Matsuyama, Ehime, Japan; ^4^ Department of Internal Medicine, Kurume University School of Medicine, Kurume, Fukuoka, Japan; ^5^ Department of Thoracic Oncology, Kanagawa Cancer Center, Yokohama, Kanagawa, Japan; ^6^ Department of Respiratory Medicine, Kitasato University School of Medicine, Sagamihara, Kanagawa, Japan; ^7^ Biostatistics Center, Kurume University School of Medicine, Kurume, Fukuoka, Japan; ^8^ Department of Pediatric Surgery, Nihon University School of Medicine, Tokyo, Japan; ^9^ Division of Cell-Free Sciences, Proteo-Science Center, PIAS, Ehime University, Matsuyama, Ehime, Japan

**Keywords:** non-small cell lung cancer, immune checkpoint inhibitor, circulating autoantibody, immune-related adverse events, immune-related pneumonitis, treatment response, machine learning

## Abstract

**Introduction:**

Immune checkpoint inhibitors (ICIs) targeting the programmed death-1/ligand-1 (PD-1/PD-L1) axis have significantly improved treatment outcomes in non-small cell lung cancer (NSCLC); however, challenges remain owing to the limited durability of therapeutic responses and the occurrence of immune-related adverse events (irAEs). This study aimed to characterize dynamic changes in the circulating autoantibody (CAAB) profile during ICI treatment and explore their association with treatment outcomes in patients with NSCLC.

**Methods:**

A panel of 59 CAABs showing substantial treatment-related changes was initially identified using AlphaScreen assays in a primary screening of five patients who developed ir-pneumonitis. These CAABs were subsequently profiled in paired pre-and post-treatment plasma samples obtained from 179 patients with NSCLC treated with anti-PD-1/PD-L1 therapy at two Japanese centers. Associations between CAAB dynamics and clinical parameters—including baseline characteristics, treatment regimens, and treatment outcomes (irAEs, ir-pneumonitis, response, progression-free survival [PFS], and overall survival [OS])—were evaluated using permutational multivariate analysis of variance and univariate binary logistic and Cox regression, elastic net regularization regression, and random forest regression.

**Results:**

Using permutational multivariate analysis of variance and univariate binary logistic/Cox regression, we comprehensively assessed the global associations between CAAB dynamics and eight clinical parameters, including background factors (PD-L1 expression and treatment line), treatment regimens (chemotherapy exposure), and treatment outcomes (irAE occurrence, ir-pneumonitis development, RECIST-assessed response, PFS, and OS), indicating that chemotherapy exposure was the only significant and strong factor influencing CAAB dynamics. In patients receiving ICI monotherapy, univariate logistic or Cox regression analyses were performed to identify individual CAABs significantly associated with each outcome, highlighting both shared and distinct immunological features underlying different clinical endpoints. Through machine learning-based evaluation of the predictive potential of CAAB dynamics for five treatment outcomes across the overall cohort and six subgroups defined by three stratification variables, four optimized CAAB signatures with robust predictive performance for ICI treatment outcomes were established.

**Conclusions:**

These findings suggest the involvement of distinct immune pathways in therapeutic benefits and toxicity. Collectively, our results provide mechanistic insights into ICI-induced humoral immune regulation, highlight the potential utility of CAABs as biomarkers to enhance benefit-to-risk assessment, and guide the development of personalized immunotherapy strategies for NSCLC.

## 1 Introduction

Although immune checkpoint inhibitors (ICIs) targeting the programmed death-1/ligand-1 (PD-1/PD-L1) axis have revolutionized the therapeutic paradigms for non-small cell lung cancer (NSCLC), two major clinical challenges remain. First, resistance limits the proportion of patients who are able to achieve a durable therapeutic response. Second, a spectrum of organ-specific inflammatory toxicities, known as immune-related adverse events (irAEs), further complicate treatment management (1, 2). These issues highlight the urgent need to elucidate the precise immunomodulatory mechanisms by which ICIs crosstalk with components involved in the host immune homeostasis. Comparative analysis of the dynamic alterations in the circulating autoantibody (CAAB) repertoire, which refers to the diversity and composition of autoantibodies present in peripheral blood, preceding and following ICI administration may yield novel mechanistic insights into treatment-induced immunomodulation. Thus, elucidating the CAAB repertoire may advance our understanding of the role of humoral immunity in both therapeutic efficacy and disruption of immune homeostasis.

Current evidence in cancer immunotherapy indicates that baseline autoantibody levels and treatment-induced antibody dynamics are associated with irAE development and therapeutic efficacy in ICI therapy (3–19). Notably, small-scale clinical studies have revealed that anti-PD-1 monotherapy induces patterns of circulating antibody/B-cell/plasmablast remodeling that are different those induced by anti-cytotoxic T-lymphocyte-associated antigen 4 (CTLA4)-containing ICI regimens, although the underlying mechanisms remain elusive (15, 20). Critical unknowns persist regarding (i) the dynamics of CAAB during PD-1/PD-L1 blockade in a large patient cohort and (ii) the existence and clinical relevance of associations between temporal autoantibody profile shifts and treatment options (monotherapy or combination therapy with chemotherapy), therapeutic response, irAE incidence, and severe complications such as pneumonitis.

**Figure 1:**
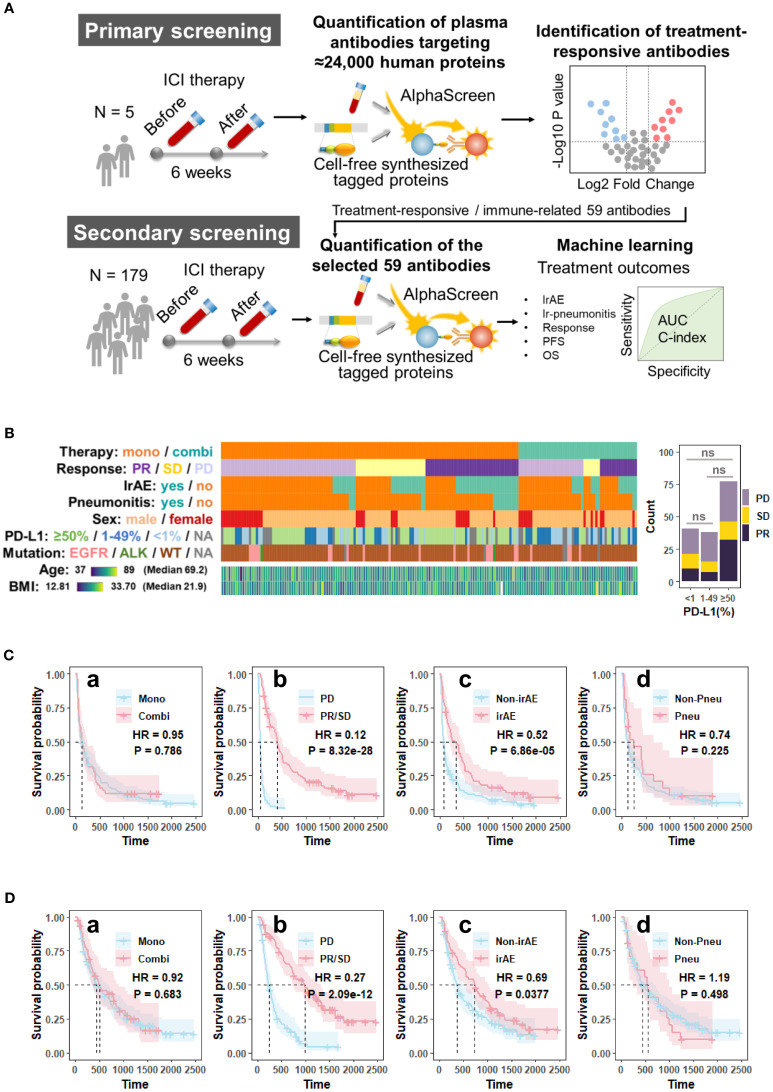
Overview of **(A)** the study design; **(B)** patient demographics, clinical characteristics, and the association between PD-L1 expression and treatment response; **(C)** Kaplan-Meier survival curves for progression-free survival (PFS); and **(D)** overall survival (OS) of the cohort (n = 179). The survival curves were generated using Cox regression based on different grouping criteria: **a** treatment option, **b** best clinical response, **c** irAE, and **d** ir-pneumonitis. ALK, anaplastic lymphoma kinase gene; AUC, area under the receiver operating characteristic curve; BMI, body mass index; Combi, combination therapy; EGFR, epidermal growth factor receptor gene; HR, hazard ratio in the Cox proportional hazards model; ICI, immune checkpoint inhibitor; irAE, immune-related adverse event; Mono, monotherapy; ns, not significant (*p* > 0.05, Chi-square test); OS, overall survival; P, p-value in the Cox proportional hazards model; PD, progressive disease; PD-L1, programmed death-ligand 1; PFS, progression-free; Pneu, ir-pneumonitis; survival; PR, partial response; SD, stable disease; WT, wild type.

In this study, we profiled 59 specific CAABs in pairs before and after treatment, using plasma samples from 179 patients who received anti-PD-1/PD-L1 therapy. By examining the antibody dynamics, we quantitatively assessed the associations between changes in the CAAB dynamic repertoire and clinical parameters, including baseline characteristics (PD-L1 expression and treatment line), treatment regimens (chemotherapy exposure), and treatment outcomes [irAE occurrence, ir-pneumonitis development, response, progression-free survival (PFS), and overall survival (OS)] (**Figure 1A**). These findings provide novel insights into the systemic immunomodulatory effects of the PD-1/PD-L1 blockade and potential autoantibody targets for disentangling therapeutic benefits from associated risks, thereby informing benefit-to-risk assessments and development of novel adjunctive strategies.

## 2 Materials and methods

### 2.1 Patients and data collection

This study included patients diagnosed with advanced, recurrent, or metastatic NSCLC who received anti-PD-1 (nivolumab or pembrolizumab) or anti-PD-L1 therapy (atezolizumab), either as monotherapy or in combination with chemotherapy, at Kurume University Hospital (Kurume, Japan) and Kanagawa Cancer Center (Yokohama, Japan). The patient cohort partially overlapped with that of a previous study conducted by our group (21, 22). The cohort consisted of 179 patients enrolled between February 2016 and August 2019. The clinical course of the enrolled patients was followed until July 2024. The patient characteristics are summarized in **Figures 1B–D**, and **Table 1**. Tumor PD-L1 expression was assessed via immunohistochemical staining of paraffin-embedded tumor sections using anti-PD-L1 monoclonal antibodies (clone E1L3N: Cell Signaling Technology, Danvers, MA, USA and clone 22C3: Agilent Technologies/Dako, Carpinteria, CA, USA). For most patients, PD-L1 expression was evaluated in tumor specimens collected prior to first-line therapy. This analysis was also done in patients who received ICIs as second- or subsequent-line treatment. Clinical response was evaluated based on the Response Evaluation Criteria in Solid Tumors (RECIST) version 1.1. IrAEs were defined according to the Common Terminology Criteria for Adverse Events (CTCAE) v5.0 grading scale. This study was conducted in accordance with the principles of the Declaration of Helsinki and was approved by the Institutional Review Boards of Kurume University Hospital (approval numbers: 15210 and 19240) and Kanagawa Cancer Center (approval number: 28-85). Informed consent was obtained from all enrolled patients after the nature of the study and its possible consequences were explained.

**Table 1:** Summary of patient characteristics in the study.

Characteristic	Value
Age (years), mean (SD)	69.2 (8.0)
Sex, n (%)	
Female	44 (24.6)
Male	135 (75.4)
BMI, mean (SD)	21.9 (3.5)
Smoking, n (%)	
Former	143 (79.9)
Never	36 (20.1)
Stages, n (%)	
Stage III	32 (17.9)
Stage IV	99 (55.3)
Recurrence	48 (26.8)
Histology, n (%)	
Non-squamous	127 (71.0)
Squamous	52 (29.0)
Driver mutation, n (%)	
Wild type	150 (83.8)
EGFR	22 (12.3)
ALK	2 (1.1)
Unknown	5 (2.8)
Tumor PD-L1 expression, n (%)	
< 1%	41 (22.9)
1% – 49%	38 (21.2)
≥ 50%	77 (43.0)
Unknown	23 (12.8)
ECOG performance status, n (%)	
0	76 (42.5)
1	71 (39.7)
2	26 (14.5)
3	6 (3.4)
Treatment line, n (%)	
First line	54 (30.2)
Further lines	125 (69.8)
Prior therapy, n (%)	
Chemotherapy	107 (59.8)
Chemotherapy and surgery	2 (1.1)
Chemotherapy and radiation	13 (7.3)
Surgery	3 (1.7)
Radiation	6 (3.4)
None	48 (26.8)
Treatment regimen, n (%)	
Monotherapy	128 (71.5)
Combination therapy	51 (28.5)
Best clinical response (RECIST), n (%)	
Partial response	56 (31.3)
Stable disease	37 (20.7)
Progressive disease	86 (48.0)
Progression-free survival (days), median (95% Cl)	131 (90–182)
Overall survival (days), median (95% CI)	468 (385–676)
Occurrence of irAE, n (%)	
None	110 (61.4)
Yes	69 (38.6)
ir-pneumonitis	21 (11.7)

ALK, anaplastic lymphoma kinase; BMI, body mass index; ECOG, Eastern Cooperative Oncology Group; EGFR, epidermal growth factor receptor; irAE, immune-related adverse event; RECIST, Response Evaluation Criteria in Solid Tumors.

### 2.2 Plasma antibody profiling

Peripheral blood samples were collected in heparin-coated tubes before and 6 weeks after therapy initiation. Plasma was separated from whole blood via centrifugation and stored at −80 °C until analysis. Plasma autoantibodies were assessed via the AlphaScreen assay using two human protein arrays generated using a wheat germ cell-free protein production system (23, 24). The HuPEX protein array, containing 19,713 human proteins, was purchased from CellFree Science Co., Ltd. (Yokohama, Japan). The Ehime-Kazusa protein array, consisting of 4,144 human proteins, was prepared in-house using a cell-free protein synthesis reagent. Gene resources for the protein array were provided by the Kazusa DNA Research Institute (Kisarazu, Japan) (25). Each cDNA clone was subcloned into the pEU-E01-FLAG-GST-K1–02 vector (26). *In vitro* transcription and translation were performed using the WEPRO7240 Expression Kit (CellFree Science) as previously described (27, 28). Briefly, the DNA fragments coding for each protein were amplified via PCR and used as templates for *in vitro* transcription. The mRNA generated by *in vitro* transcription was used as a template for *in vitro* translation (total reaction mixture, 5 µL; components, 2.5 µL mRNA; 1.67 µL WEPRO 7240 wheat germ extract; 0.14 µL creatine kinase [20 mg/mL]; and 0.11 µL RNase inhibitor) was layered below 50 µL SUB-AMIX SGC substrate solution in a 384-well plate and incubated at 20°C for 18 h. The translated protein arrays were diluted 2-fold with AlphaScreen buffer (100 mM Tris-HCl, pH 8.0; 0.01% Tween 20; 1 mg/mL BSA), aliquoted into 384-well plates, flash-frozen in liquid nitrogen, and stored at −80°C.

The AlphaScreen assay was performed as previously described, with minor modifications (27). Specificity was achieved through two independent high-affinity binding steps (1): glutathione-coated donor beads that selectively bind GST-tagged recombinant antigens, and (2) Protein G–conjugated acceptor beads that specifically recognize human IgG, thereby minimizing nonspecific interactions. For primary screening, autoantibody reactivity was assessed after incubating 10 paired plasma samples from patients with NSCLC (five pre- and post-ICI treatment, respectively) with a library of 23,857 human proteins. Plasma samples were diluted 1:667 in AlphaScreen reaction buffer (100 mM Tris-HCl, pH 8.0, 0.01% [v/v] Tween 20, 0.1% [w/v] bovine serum albumin), and 20 µL diluted plasma was dispensed into each well of OptiPlate-384 plates (Revvity, Yokohama, Japan) using a Liquidator96 pipetting system. Next, 1 µL of each FLAG-GST tagged protein was transferred from 384-well stock plate to the reaction plate using JANUS automated dispensing workstation equipped with a NanoHead 384-channel microsyringe head (Revvity). Subsequently, 9 µL detection mixture (containing 0.06 µL AlphaScreen GSH Donor Beads [Revvity] and 0.06 µL Protein G-conjugated AlphaScreen acceptor beads in reaction buffer) was added to the reaction plates with a FlexDrop dispenser (27). After incubation at 25°C for 1 h in the dark, AlphaScreen signals were detected using an EnVision plate reader (Revvity).

For secondary screening, 358 paired plasma samples from 179 patients with NSCLC (pre- and post-treatment) were tested for the 59 selected proteins, each in quadruplicate. All reactions were performed on AlphaPlate-1536 plates (Revvity). Three microliters of protein dilution containing 0.05 µL FLAG-GST tagged protein was dispensed into an AlphaPlate 1536 plate (Revvity) via a Multidrop Combi nL (Thermo Fisher Scientific, Tokyo, Japan). All 358 plasma samples were diluted 1:40 and dispensed into a 384 well plate. Subsequently, 0.2 µL of the 1:40 diluted plasma from each well of these 384-well plates was dispensed into the 1536-well reaction plates via a JANUS workstation with a NanoHead. Finally, 1.8 µL detection mixture (containing 0.01 µL AlphaScreen GSH Donor Beads and 0.01 µL Protein G-conjugated AlphaScreen acceptor beads) was added to the reaction plates via a Multidrop Combi nL. After incubation at 25°C for 1 h in the dark, AlphaScreen signals were detected with an EnVision plate reader. The secondary screening minimized inter-plate variability by ensuring that paired plasma samples (pre- and post-ICI) from the same patient were analyzed on the same plate. To further minimize experimental variability, all assays were conducted on a single 1536-well plate uniformly coated with the same antigen. Plasma samples from all patients were dispensed in quadruplicate. This design enabled direct assessment of treatment-induced changes both within individual patients and across the cohort, while controlling for inter-plate variability.

### 2.3 Machine learning and statistical analysis

All analyses were performed using R (version 4.4.1; https://www.r-project.org). Univariate binary logistic and Cox proportional hazards regression analyses were performed using the glm function (R Basic package). P-values were derived using Wald z-tests and subsequently adjusted for multiple comparisons using the Benjamini–Hochberg false discovery rate (FDR) procedure. To mitigate overfitting in multivariate predictive modeling, we applied elastic net (EN) regularized regression and random forest (RF) regression for binary outcome variables (irAE, ir-pneumonitis, and treatment response), and Cox proportional hazards regression with elastic net regularization (EN-Cox) and random survival forest (RSF) for survival outcomes (PFS and OS) within a machine learning framework (21). The cohort was randomly stratified into training (80%) and validation (20%) groups. Model development and hyperparameter optimization were conducted using nested cross-validation (CV) exclusively on the training set. The final models were subsequently retrained on the entire training set and independently evaluated on the validation set, maintaining a strict separation between the development and evaluation datasets to prevent data leakage. For EN regression, hyperparameter tuning (α = 0.5 for balanced L1/L2 regularization) was performed using 10-fold CV via the cv.glmnet implementation (glmnet package), optimizing the regularization parameter λ to minimize classification error. The λ value yielding minimum CV error was selected for final model training, followed by external validation on the holdout set. For the RF regression, feature selection was performed via sequential variable inclusion based on importance rankings, with variable importance assessed using the mean Gini index (randomForest package). The optimal feature subset (important factors) was identified by maximizing predictive accuracy during stepwise variable addition. Model performance was assessed using leave-one-out cross-validation (LOO-CV) of the training set with discriminatory power quantified by the area under the receiver operating characteristic curve (AUC). All RF implementations utilized an ensemble of 1,000 decorrelated decision trees with the minimum node purity set to 1, following established methodologies in high-dimensional regression (29). Optimization of the model cutoff value was performed by maximizing Youden’s *J* statistic (sensitivity + specificity - 1), implemented through the coords function in R. Permutational multivariate analysis of variance (PERMANOVA) was performed using the Adonis function (vegan package). Nonparametric comparisons were performed using the Wilcoxon signed-rank test via the wilcox.test function. Survival analyses included Kaplan–Meier curve generation with log-rank testing, alongside Cox proportional hazards modeling, executed using the survival and survminer packages in R.

## 3 Results

### 3.1 Patient characteristics

As shown in **Figure 1** and **Table 1**, of the 179 patients with NSCLC included in this study, 128 (71.5%) received anti-PD-1/PD-L1 monotherapy and 51 (28.5%) received combination therapy with chemotherapy. The best overall response was partial response (PR) in 56 patients (31.3%), stable disease (SD) in 37 patients (20.7%), and progressive disease (PD) in 86 patients (48.0%). IrAEs occurred in 69 patients (38.6%) and ir-pneumonitis was observed in 21 patients (11.7%). The median PFS in the cohort was 131 days and the median OS was 468 days. **Figure 1C** illustrates PFS across the clinical subgroups. No significant differences in PFS were observed between patients who received combination therapy and those who received monotherapy (hazard ratio [HR] = 0.95, *p* = 0.786). Patients in the responder group (PR + SD) demonstrated significantly prolonged PFS compared to those in the PD group (HR = 0.12, *p* < 0.001). Similarly, patients who developed irAEs had a longer PFS than those who did not (HR = 0.52, *p* < 0.001). However, patients with ir-pneumonitis did not show a significant difference in PFS compared to those without ir-pneumonitis (HR = 0.74, *p* = 0.225). **Figure 1D** shows OS data across the same subgroups. Consistent with the PFS results, the OS did not differ significantly between the ICI monotherapy and combination therapy (HR = 0.92, *p* = 0.683) groups. The responder group exhibited significantly improved OS compared with the PD group (HR = 0.27, *p* < 0.001). Additionally, patients with irAEs had a longer OS than those without irAEs (HR = 0.69, *p* = 0.0377). In contrast, patients who developed ir-pneumonitis showed no OS benefit from treatment (HR = 1.19, *p* = 0.498).

### 3.2 Circulating autoantibody titers exhibit a predominant decrease during PD-1/PD-L1 blockade

For primary screening, we used AlphaScreen to quantify plasma IgG antibodies against approximately 24,000 biotinylated proteins in pre- and post-treatment plasma from five patients with NSCLC who developed ir-pneumonitis during ICI treatment. Based on pre/post signal ratios, 37 antibodies showed significant changes (*p* < 0.05, Student’s *t*-test; fold changes < 0.7 or > 1.4). To offset the limited sample size, 22 additional antibodies were included due to notable fold changes and prior associations with immune-related pathways, cancer biology, or ICI responses reported in the literature. In total, 59 antibodies were selected for further analysis (**Supplementary Table S1**).

**Figure 2:**
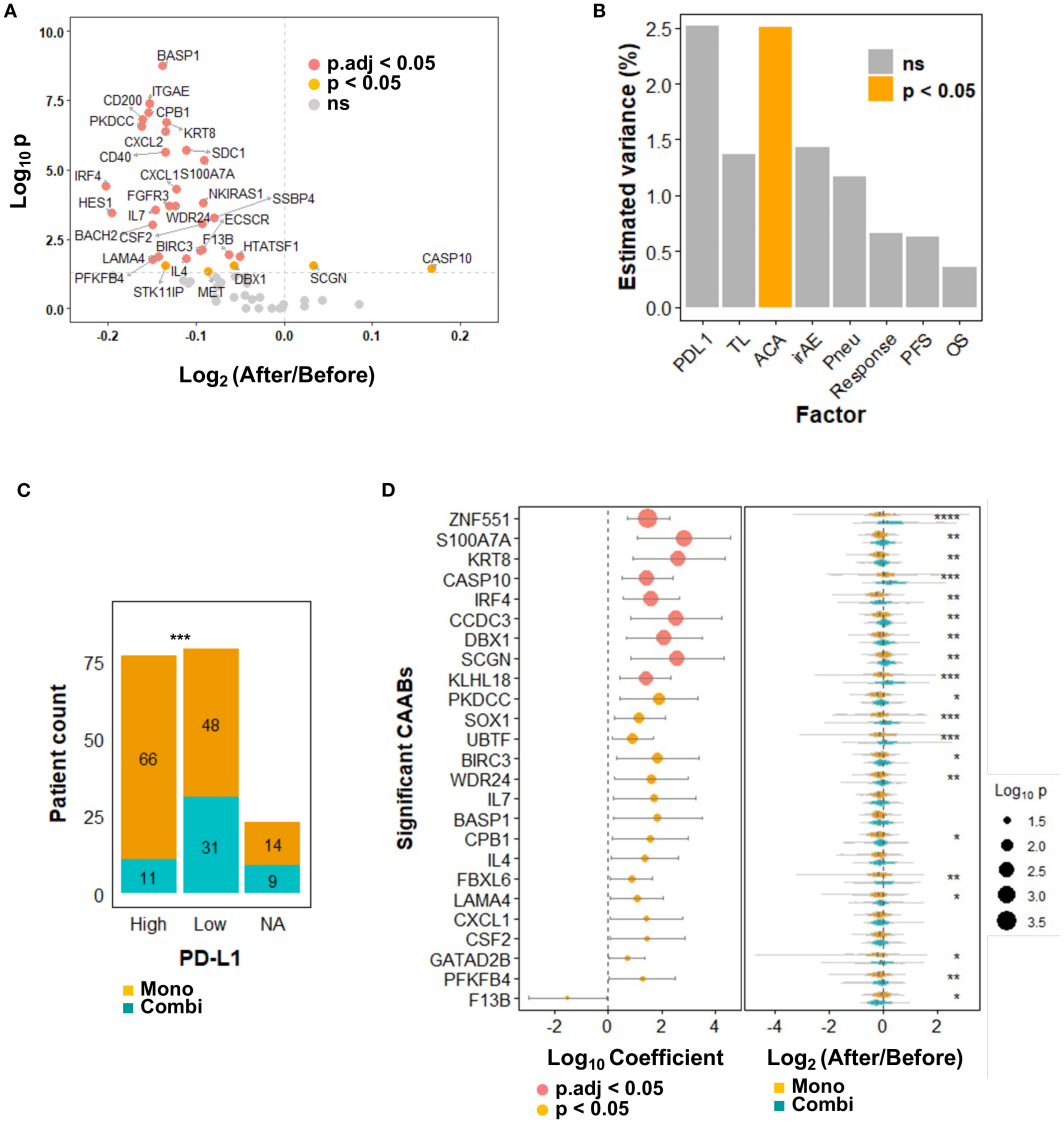
Dynamics of 59 protein-specific circulating autoantibodies (CAABs) before and after ICI therapy (n = 179). **(A)** Volcano plot illustrating differential antibody responses: Wilcoxon signed-rank test p-values; *p.adj* indicates false discovery rate (FDR) correction using the Benjamini-Hochberg method. **(B)** PERMANOVA analysis quantifying inter-individual variation in CAAB dynamics explained by background factors (PD-L1 expression and treatment line), treatment regimens (monotherapy vs. combination therapy), and treatment outcomes (irAE occurrence, ir-pneumonitis development, RECIST-assessed response, PFS, and OS). Chemotherapy exposure emerged as the only significant (*p* < 0.05) modulator of CAAB dynamics among ICI-related clinical parameters; no factor showed *p.adj* < 0.05. **(C)** Association between PD-L1 expression (≥ 50%: High; < 50%: Low) and treatment options in the present cohort: Chi-square test p-values; ***p < 0.001. **(D)** Univariate logistic regression evaluating chemotherapy-associated CAABs: Wald test p-values; all significant CAABs (*p* < 0.05) are displayed; *p.adj* indicates Benjamini-Hochberg-adjusted FDR. Violin plots demonstrate group-wise comparisons using Wilcoxon rank-sum test significance levels: ****p < 0.0001; ***p < 0.001; **p < 0.01; *p < 0.05. ACA, anticancer agent; Combi, combination therapy; FDR, false discovery rate; ICI, immune checkpoint inhibitor; irAE, immune-related adverse event; Mono, monotherapy; NA, not available; ns, not significant; OS, overall survival; PERMANOVA, permutational multivariate analysis of variance; PFS, progression-free survival; Pneu, ir-pneumonitis; TL, treatment line.

In secondary screening, these 59 candidates were quantified in pre- and post-treatment plasma from a formal cohort of 179 patients. A volcano plot (**Figure 2A**) illustrates changes in CAAB levels before and after anti-PD-1/PD-L1 therapy: 53 decreased (average log_2_-transformed fold-change < 0), 30 significantly (*p* < 0.05), including BASP1, ITGAE, CPB1, and CD200 autoantibodies. Six increased (average log_2_-transformed fold-change > 0), with two autoantibodies against CASP10 and SCGN reached statistical significance.

### 3.3 Chemotherapy exposure as the predominant determinant of circulating autoantibody repertoire changes

We calculated the log_2_-transformed fold-change for each CAAB, designating this dataset as the dynamic repertoire. Clinical relevance of the CAAB dynamic repertoire by computing multivariate effect sizes for treatment regimen, treatment line, PD-L1 expression, occurrence of irAE and ir-pneumonitis, therapeutic response, PFS, and OS using PERMANOVA (30). As shown in **Figure 2B**, the CAAB dynamic repertoire exhibited a significant association only with chemotherapy exposure among all clinical factors assessed (highest R² = 2.5%, *p* < 0.05), indicating marked sensitivity to anticancer agent administration. Regarding clinical baseline characteristics, CAAB dynamics demonstrated a stronger effect size with PD-L1 expression than with the line of therapy, although without significance. In the present cohort, the therapeutic decisions (monotherapy versus combination therapy) were directly influenced by PD-L1 expression (**Figure 2C**) (31). Among the five treatment outcome measures—irAEs, ir-pneumonitis, RECIST-assessed response, PFS, and OS—the effect size of the association with CAAB dynamics decreased in the following order: irAEs, ir-pneumonitis, response, PFS, and OS, although none reached statistical significance (**Figure 2B**). To further elucidate which CAABs exhibited the strongest association with concomitant anticancer agent administration during ICI therapy, we performed univariate logistic regression analyses stratified by anticancer agent use. CAABs with a significance threshold of *p* < 0.05 in the regression analyses are summarized in **Figure 2D**. Nine CAABs retained statistical significance after FDR correction: antibodies against ZNF551, S100A7A, KRT8, CASP10, IRF4, CCDC3, DBX1, SCGN, and KLHL18. Several other CAABs exhibited notable associations, including PKDCC, SOX1, UBTF, BIRC3, WDR24, IL7, BASP1, CPB1, IL4, FBXL6, LAMA4, CXCL1, CSF2, GATAD2B, PFKFB4, and F13B. Plasma levels of these autoantibodies were significantly modulated by anticancer agent coadministration. Violin plots (**Figure 2D**) illustrate distinct expression patterns. ZNF551-targeting CAABs decreased after monotherapy but increased with combination therapy. Similarly, S100A7A and KRT8 CAABs decreased with monotherapy, but remained stable before and after combination therapy. CASP10 autoantibody levels increased in both groups, albeit less markedly in monotherapy.

### 3.4 Circulating autoantibody dynamics associated with treatment outcomes under anti-PD-1/PD-L1 monotherapy

**Figure 3:**
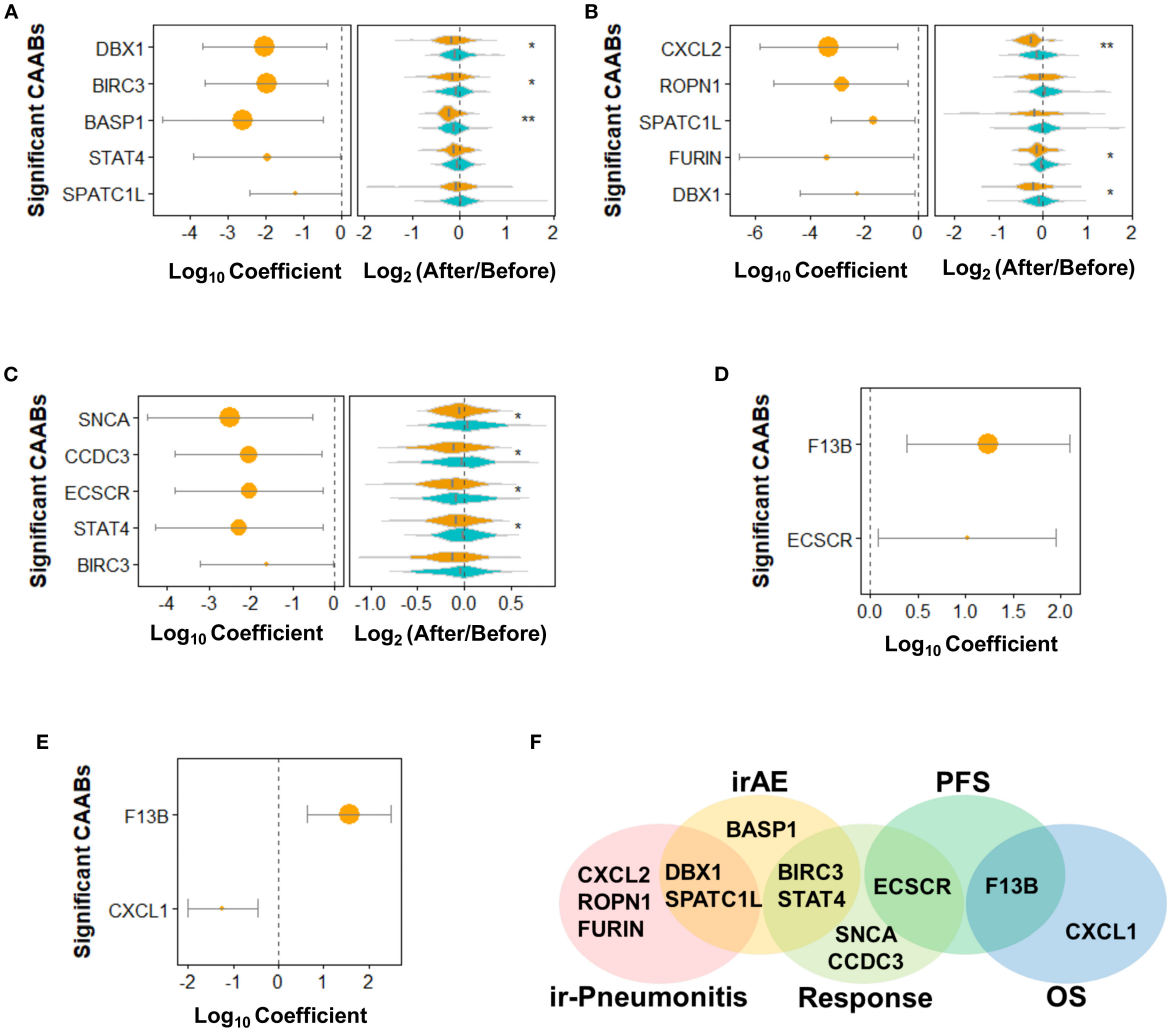
Dynamics of 59 protein-specific circulating autoantibodies (CAABs) during ICI therapy in patients receiving monotherapy (n = 128). Univariate logistic regression was used to evaluate **(A)** irAE-, **(B)** ir-pneumonitis-, and **(C)** RECIST-assessed response-associated CAABs; univariate Cox regression was used to evaluate **(D)** PFS- and **(E)** OS-associated CAABs. All significant CAABs (*p* < 0.05, Wald test) are displayed; no CAABs showed. *p.adj* < 0.05 after Benjamini-Hochberg FDR adjustment. Violin plots demonstrate group-wise comparisons using Wilcoxon rank-sum test significance levels: **p < 0.01; *p < 0.05. **(F)** Summary Venn diagram of CAABs significantly associated with irAE, ir-pneumonitis, response, PFS, and OS. FDR, false discovery rate; ICI, immune checkpoint inhibitor; irAE, immune-related adverse event.

Through the above analyses, we identified that the treatment regimen was the primary significant factor influencing the dynamic repertoire of CAAB during ICI therapy. Given the non-negligible influence of anticancer agents, we further examined the association between CAAB fluctuation patterns and treatment outcomes in a subgroup of patients receiving ICI monotherapy. To identify the autoantibodies specifically associated with each treatment outcome (irAEs, ir-pneumonitis, treatment response, PFS, and OS), we performed individual univariate logistic/Cox regression analyses for each factor. **Figure 3A** shows that in patients receiving ICI monotherapy, CAABs targeting DBX1, BIRC3, BASP1, STAT4, and SPATC1L were significantly associated with the occurrence of irAEs. The irAE-positive subgroup exhibited more pronounced post-treatment reductions in autoantibody titers than the controls. **Figure 3B** presents the univariate regression analyses of pneumonitis-associated CAABs, revealing significant correlations for autoantibodies targeting CXCL2, ROPN1, SPATC1L, FURIN, and DBX1. Consistent with the irAE pattern, patients with ir-pneumonitis showed greater treatment-related decreases in CAABs, with CXCL2 showing the greatest reduction. Regarding the therapeutic efficacy, response-associated CAABs included autoantibodies against SNCA, CCDC3, ECSCR, STAT4, and BIRC3 (**Figure 3C**). The treatment responders displayed a significantly greater absolute reduction in CAAB levels after ICI administration. PFS- and OS-associated CAABs included autoantibodies against F13B, ECSCR, and CXCL1 (**Figures 3D, E**). The Venn diagram in **Figure 3F** illustrates both shared and unique CAABs across different treatment outcomes. Autoantibodies against BIRC3 and STAT4 were common to irAEs and treatment responses, whereas SPATC1L and DBX1 were associated with both irAEs and ir-pneumonitis. Treatment response was characterized by distinct autoantibodies targeting SNCA, CCDC3, and ECSCR, with ECSCR additionally shared with PFS. Autoantibodies against CXCL1 were specifically associated with OS, while ir-pneumonitis showed unique associations with CXCL2, ROPN1, and FURIN, highlighting the differential CAAB dynamics underlying these outcomes.

### 3.5 Machine learning for prediction of treatment outcomes using circulating autoantibody dynamics

**Figure 4:**
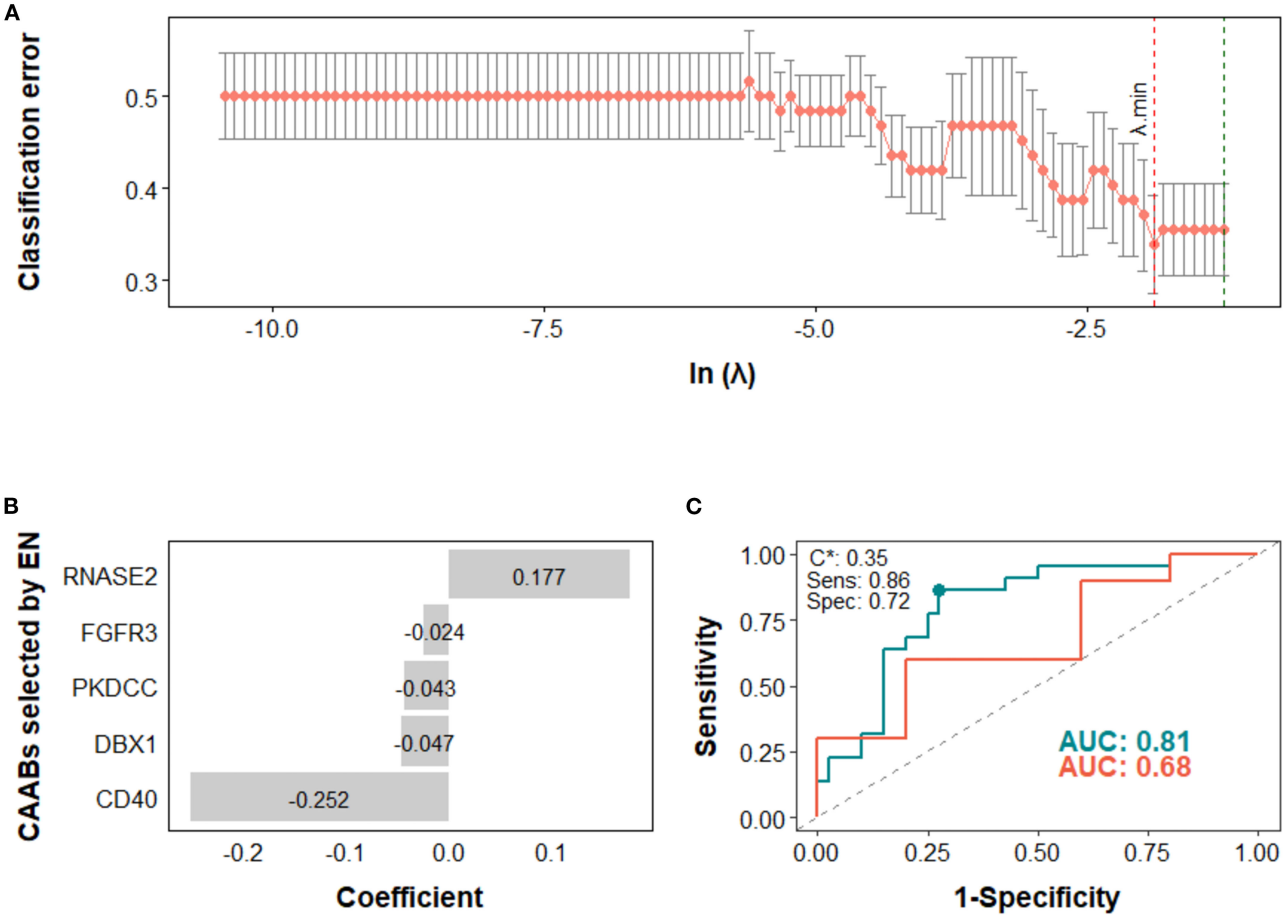
Predictive modeling of irAE occurrence using dynamic profiles of circulating autoantibodies (CAABs) in the high PD-L1 subgroup (n = 77). In the elastic-net (EN) regression-based models: **(A)** the penalty hyperparameter (λ) was determined through 10-fold cross-validation (CV) across a range of λ values; the optimal λ (λ.min) corresponding to the minimum CV classification error was selected. **(B)** CAABs selected by EN at λ.min. **(C)** ROC curves derived from the training and test sets, with AUCs of 0.81 and 0.68, respectively; the model threshold (C*) was optimized using the maximum Youden’s *J* statistic, yielding a sensitivity of 0.86 and specificity of 0.72. AUC, area under the receiver operating characteristic curve; CV, cross-validation; EN, elastic-net; ROC, receiver operating characteristic curve; Sens, sensitivity; Spec, specificity.

**Figure 5:**
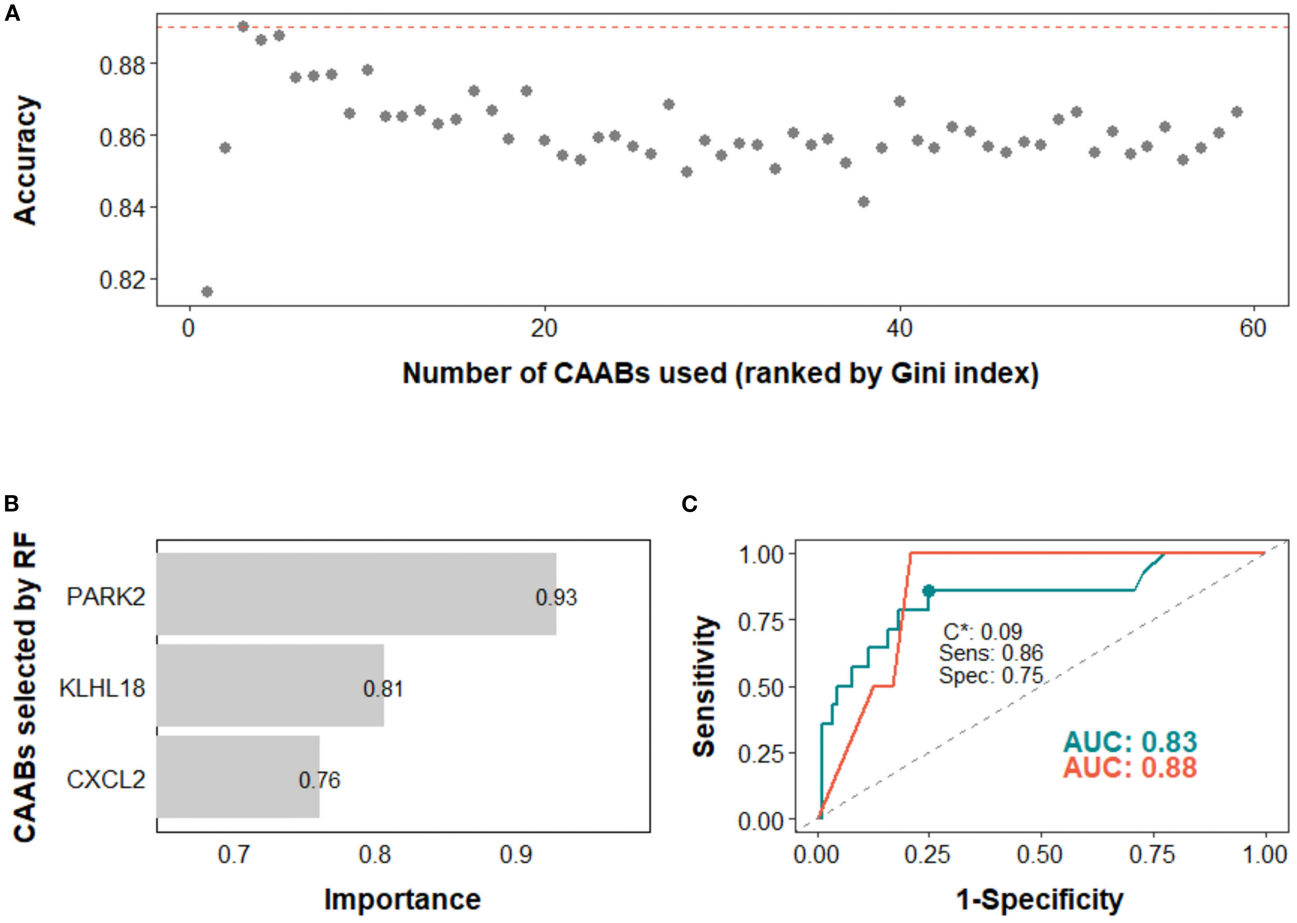
Predictive modeling of ir-pneumonitis occurrence using dynamic profiles of circulating autoantibodies (CAABs) in the monotherapy subgroup (n = 128). In the random forest (RF)-based models: **(A)** predictive accuracy during stepwise addition of CAABs according to their importance ranked by the Gini index; the RF model using the top three CAABs achieved the highest predictive accuracy. **(B)** CAABs selected by the RF model. **(C)** ROC curves derived from the training and test sets, with AUCs of 0.83 and 0.88, respectively; the model threshold (C*) was optimized using the maximum Youden’s *J* statistic, yielding a sensitivity of 0.86 and a specificity of 0.75. AUC, area under the receiver operating characteristic curve; RF, random forest; ROC, receiver operating characteristic curve; Sens, sensitivity; Spec, specificity.

**Figure 6:**
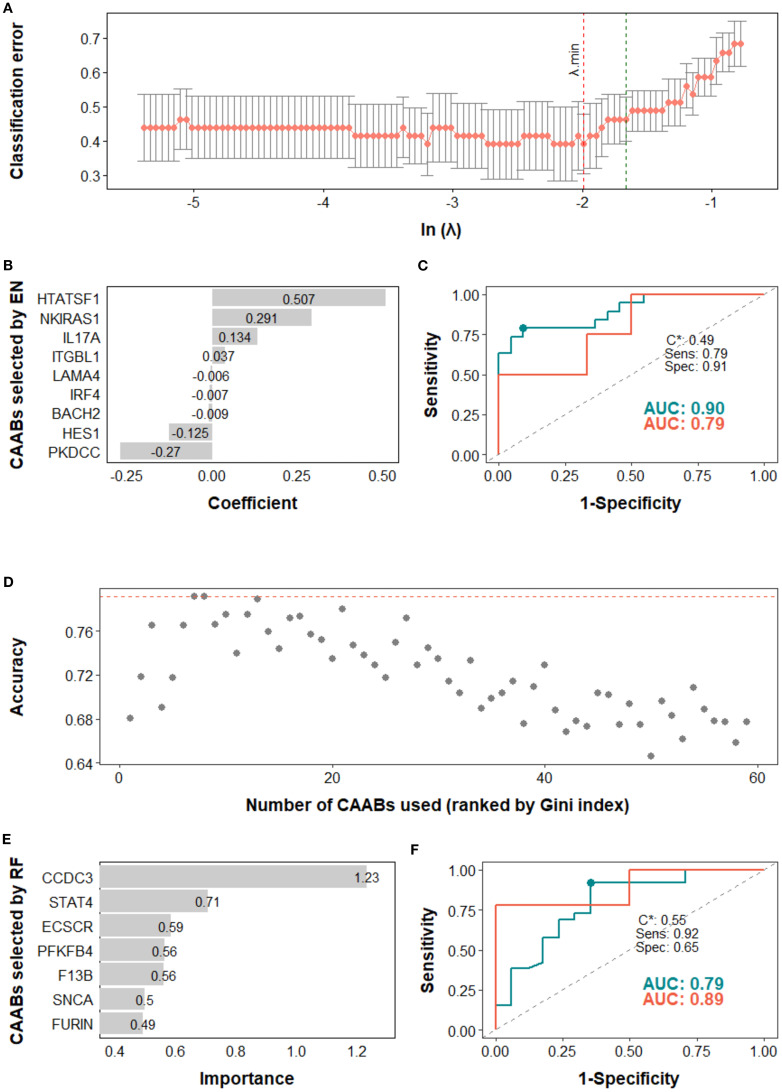
Predictive modeling of treatment response using dynamic profiles of circulating autoantibodies (CAABs) in the **(A–C)** combination therapy subgroup (n = 51) and **(D–F)** first line subgroup (n = 54). In the elastic-net (EN) regression-based models of combination therapy subgroup: **(A)** the penalty hyperparameter (λ) was determined through 10-fold cross-validation (CV) across a range of λ values; the optimal λ (λ.min) corresponding to the minimum CV classification error was selected. **(B)** CAABs selected by EN at λ.min. **(C)** ROC curves derived from the training and test sets, with AUCs of 0.90 and 0.79, respectively; the model threshold (C*) was optimized using the maximum Youden’s *J* statistic, yielding a sensitivity of 0.79 and specificity of 0.91. In the random forest (RF)-based models of first line subgroup: **(D)** predictive accuracy during stepwise addition of CAABs according to their importance ranked by the Gini index; the RF model using the top seven CAABs achieved the highest predictive accuracy. **(E)** CAABs selected by the RF model. **(F)** ROC curves derived from the training and test sets, with AUCs of 0.79 and 0.89, respectively; the model threshold (C*) was optimized using the maximum Youden’s *J* statistic, yielding a sensitivity of 0.92 and a specificity of 0.65. AUC, area under the receiver operating characteristic curve; CV, cross-validation; EN, elastic-net; RF, random forest; ROC, receiver operating characteristic curve; Sens, sensitivity; Spec, specificity.

Next, we assessed the potential of CAAB as a predictive biomarker for treatment outcomes associated with anti-PD-1/PD-L1–based ICI therapy. The predictive performance of CAAB dynamics for five treatment outcomes across the overall cohort and six subgroups defined by three stratification variables is summarized in **Table 2**. As shown, models using the entire cohort without stratification demonstrated limited predictive accuracy. In contrast, stratified analyses revealed notable performance: for irAE prediction, the EN model in the high PD-L1 subgroup attained AUCs of 0.81 and 0.68 for the training and test sets, respectively (**Figure 4**); for ir-pneumonitis prediction, the RF model in the monotherapy subgroup achieved AUCs of 0.83 and 0.88 (**Figure 5**); for response prediction, the EN model in the combination therapy subgroup exhibited AUCs of 0.90 and 0.79; and in the first-line treatment subgroup, the RF model achieved AUCs of 0.79 and 0.89 (**Figure 6**). By contrast, the predictive capacity of CAAB dynamics for PFS and OS remained limited.

**Table 2:** Predictive performance of circulating autoantibody (CAAB) dynamics for five treatment outcomes overall and in six subgroups defined by three stratification conditions.*^a^*

Stratification factor	Subgroup	Sample number	irAE				Pneu				Response^d^				PFS				OS			
			EN^b^		RF		EN^b^		RF^c^		EN^b^		RF		EN-Cox		RSF		EN-Cox		RSF	
			tr	te	tr	te	tr	te	tr	te	tr	te	tr	te	tr	te	tr	te	tr	te	tr	te
None	-	179	0.69	0.62	0.69	0.60	—		0.69	0.66	0.79	0.50	0.70	0.57	0.50	0.44	0.53	0.46	0.54	0.55	0.54	0.50
Treatment regimen	Mono	128	0.71	0.61	0.72	0.62	—		0.83	0.88	—		0.59	0.56	0.54	0.46	0.55	0.55	0.61	0.57	0.53	0.45
	Combi	51	—		0.69	0.56	—		—		0.90	0.79	0.75	0.71	0.49	0.50	0.49	0.62	0.48	0.44	052	0.44
Treatment line	First	54	—		0.72	0.57	—		0.75	0.58	—		0.79	0.89	0.43	0.42	0.59	0.40	0.40	0.60	0.51	0.44
	Further	125	—		0.58	0.53	—		0.65	0.50	0.66	0.59	0.66	0.51	0.54	0.65	0.48	0.38	0.55	0.53	0.50	0.34
PD-L1	High (≥50%)	77	0.81	0.68	0.73	0.56	—		0.81	0.71	0.74	0.57	0.68	0.68	0.48	0.69	0.46	0.74	0.50	0.52	0.53	0.62
	Low (<50%)	79	0.71	0.52	0.72	0.51	—		0.58	0.54	0.70	0.68	0.79	0.56	0.55	0.51	0.50	0.58	0.54	0.55	0.52	0.51

^*a*^For irAE, ir-pneumonitis, and response, AUCs of the models were calculated for both the training (tr) and test (te) sets; for PFS and OS, the C-index of survival models was evaluated;

^*b*^“–” indicates model failure using the elastic-net (EN) method: the penalty hyperparameter λ resulted in a null model, in which all coefficients were shrunk to zero;

^*c*^“–” indicates model failure using the random forest (RF) method due to an insufficient number of positive cases;

^*d*^For RECIST-assessed treatment response, PR and SD were defined as responders, whereas PD was defined as non-responders.

Combi, combination therapy; EN, elastic-net; EN-Cox, Cox proportional hazards regression with elastic net regularization; irAE, immune-related adverse event; Mono, monotherapy; OS, overall survival; PD, progressive disease; PD-L1, programmed death-ligand 1; PFS, progression-free survival; Pneu, immune-related-pneumonitis; PR, partial response; RF, random forest; RSF, random survival forest; SD, stable disease.

In the high PD-L1 subgroup, the EN model selected five CAABs (RNASE2, FGFR3, PKDCC, DBX1, and CD40). The optimized cutoff value of 0.35 yielded a specificity of 0.86 and a sensitivity of 0.72 for predicting irAEs (**Figure 4**). the RF model with the top three features (PARK2, KLHL18, CXCL2) achieved the best performance, with a cutoff of 0.09 (specificity 0.86, sensitivity 0.75) for predicting ir-pneumonitis (**Figure 5**). For treatment response in the combination therapy subgroup, the EN model highlighted nine CAABs (HTATSF1, NKIRAS1, IL17A, ITGBL1, LAMA4, IRF4, BACH2, HES1, PKDCC), with a cutoff of 0.49 (specificity 0.79, sensitivity 0.91) (**Figures 6A–C**). In the first-line treatment subgroup, the RF model selected seven CAABs (CCDC3, STAT4, ECSCR, PFKFB4, F13B, SNCA, FURIN), with a cutoff of 0.55 (specificity 0.92, sensitivity 0.65) (**Figures 6D–F**). Both models demonstrated favorable predictive performance for treatment response within their respective subgroups.

These results indicate that patient background and treatment options distinctly shape CAAB dynamics, and that capturing subset-specific dynamic signatures may enable effective prediction of treatment outcomes across different clinical contexts or therapeutic regimens.

## 4 Discussion

Characterizing the dynamic changes in CAAB profiles during ICI therapy provides critical insights into the immunomodulatory effects of ICIs on host immunity. In this study, we initially identified 59 autoantibodies that exhibited substantial alterations during anti-PD-1/PD-L1 treatment in a primary screening of peripheral blood samples from five patients with NSCLC who developed ir-pneumonitis following ICI therapy. These candidate CAABs were subsequently quantified in plasma samples from an expanded cohort of 179 patients. PERMANOVA was applied to comprehensively assess the global associations between CAAB dynamics and eight clinical parameters, including background factors (PD-L1 expression and treatment line), treatment regimens (chemotherapy exposure), and treatment outcomes (irAE occurrence, ir-pneumonitis development, RECIST-assessed response, PFS, and OS). For patients receiving ICI monotherapy, univariate logistic or Cox regression analyses were performed to identify individual CAABs significantly associated with each treatment outcome factor. Finally, within a machine learning framework incorporating rigorous robustness evaluations, we examined the predictive potential of CAAB dynamics for five treatment outcomes in the overall cohort and across six subgroups defined by three stratification variables, and established four optimized CAAB signatures relevant to ICI treatment outcomes. Among all clinical factors assessed, the CAAB dynamic repertoire showed a significant association solely with chemotherapy exposure, highlighting its pronounced sensitivity to anticancer drug administration. Several mechanisms may explain this phenomenon. For example, cytotoxic anticancer agents can induce immunogenic cell death, such as apoptosis and necrosis, in both tumor and healthy cells, resulting in the release of self-antigens. This antigen release may enhance antigen presentation by antigen-presenting cells and promote a pro-inflammatory environment, ultimately contributing to the breakdown of immune tolerance and activation of autoreactive lymphocytes. In addition, some anticancer agents have been reported to reduce immunosuppressive cell populations, including regulatory T cells and myeloid-derived suppressor cells (MDSCs), thereby facilitating the production of autoantibodies (32). CAAB dynamics also demonstrated a relatively strong, albeit non-significant, correlation with PD-L1 expression. Considering the substantial influence of PD-L1 status on treatment options in the present study cohort, further investigations are warranted to clarify the potential association between PD-L1 expression and CAAB dynamics during ICI therapy.

In the monotherapy group, univariate logistic and Cox regression analysis revealed associations between CAAB dynamics and treatment outcomes during ICI therapy, highlighting both shared and distinct immunological features underlying different clinical endpoints. Although irAEs have been proposed as a potential clinical marker of ICI responsiveness, the exact nature and extent of this relationship remain incompletely understood (1, 33–36). This biological distinction underscores the importance of methodologically separating irAE-related signals from those linked to therapeutic efficacy, and highlighting the need for comprehensive benefit-to-risk assessment to guide clinical decision-making (37). Notably, CAABs against BIRC3 and STAT4 were associated with both treatment response and irAE occurrence, suggesting that the underlying immune mechanisms linked to these antibodies may contribute to therapeutic efficacy, while also driving adverse immune activation. In contrast, CAABs against SNCA, CCDC3, and ECSCR were significantly associated only with treatment response, indicating the possible existence of tumor-specific immune regulatory pathways independent of irAEs. These findings offer a novel perspective for disentangling treatment efficacy from immune-related toxicity, thereby supporting optimized benefit-to-risk assessments in clinical practice.

In addition, we observed that the majority of selected CAABs showed decreased plasma concentrations following ICI treatment. Previous studies have reported that blocking PD-1/PD-L1 enhances the maturation of PD-1-expressing T follicular helper (Tfh) cells on B cells, thereby augmenting antibody production against exogenous antigens (38, 39). However, clinical data suggest divergent effects depending on the ICI class. In a cohort of 39 patients, anti-CTLA-4 monotherapy or CTLA-4–containing combinations were associated with increased circulating plasmablasts (CD38^+^CD27^+^), whereas anti-PD-1 monotherapy was linked to reduced plasmablast numbers (20). Similarly, a study involving 48 patients reported that anti-CTLA-4 treatment generally led to increased CAAB levels, whereas anti-PD-L1 treatment tended to reduce them (15). More recently, statistical analyses of CAABs in 102 patients corroborated these trends (16). Although the mechanisms underlying these observations are poorly understood, these divergent outcomes may partly reflect the distinct sites and timing of immune modulation. CTLA-4 inhibition acts primarily during the priming phase within secondary lymphoid organs, promoting polyclonal B-cell activation, including autoreactive clones, and sustaining plasmablast output and survival (9, 20). In contrast, PD-1/PD-L1 blockade acts mainly within germinal centers and peripheral tissues, enhancing the quality of Tfh–mediated B-cell help rather than inducing global plasmablast expansion, and favoring transient expansion of high-affinity, antigen-specific clones (38). Such responses may wane once the antigenic stimulus declines. Another speculative mechanism is local immune-complex formation within tumor or inflamed tissues, which could promote antibody sequestration or consumption, thereby lowering circulating titers despite ongoing local humoral activity. However, these tissue-level antibody dynamics have not been directly demonstrated in the context of PD-1 versus CTLA-4 blockade, meriting further mechanistic investigation.

The best-performing model for predicting ir-pneumonitis was the RF model in the monotherapy subgroup. Identified CAABs targeting CXCL2, PARK2, and KLHL18 may contribute to pathogenic immune hyperactivation, thus warranting further investigation. Our previous findings indicated that decreased plasma CXCL2 levels after ICI treatment are associated with irAE occurrence in patients with NSCLC (40). CXCL2 promotes the recruitment of MDSCs via CXCR2-mediated signaling, thereby contributing to the establishment of an immunosuppressive microenvironment (41, 42). In contrast, reduced CXCL2 levels may reflect a shift toward a pro-inflammatory milieu. Among patients who developed ir-pneumonitis, we observed lower levels of CXCL2-specific CAABs. This reduction may reflect diminished antigen-driven antibody production or sequestration within CXCL2–antibody complexes, resulting in lower detectable plasma levels. Further work is required to test these hypotheses and clarify their relevance to irAE pathogenesis.

The overlapping associations of BIRC3 and STAT4 with both therapeutic efficacy and the occurrence of irAEs, together with the selective link of CXCL2 with pneumonitis, suggest the existence of a shared yet bi-directional immunoregulatory axis. BIRC3, a regulator of nuclear factor kappa-light-chain-enhancer of activated B cell (NF-κB)-dependent transcription, and STAT4, a mediator of IL-12-driven type 1 helper T cell (Th1) polarization and type II interferon (IFN-γ) production, can amplify systemic pro-inflammatory signaling that enhances anti-tumor immunity while heightening susceptibility to multi-organ irAEs (43, 44). In contrast, CXCL2 recruits CXCR2^+^ MDSCs, providing a counter-regulatory brake on inflammation, with pulmonary tissue particularly dependent on this chemokine axis (45). In our cohort, reductions in BIRC3- and STAT4-specific autoantibodies were associated both with enhanced therapeutic efficacy and increased irAEs. One plausible explanation is that early activation of NF-κB/Th1 programs transiently augments antigen presentation and autoantibody production, followed by antigen clearance or a shift toward cell-mediated immunity, leading to lower autoantibody titers despite sustained pathway activity. By contrast, decreases in CXCL2-specific autoantibodies were unrelated to efficacy but correlated with pneumonitis, consistent with the idea that disrupting the CXCL2–CXCR2 axis removes a local anti-inflammatory safeguard in the lung, creating a predisposition to organ-restricted toxicity without broadly influencing systemic anti-tumor immunity (45). These findings are hypothesis-generating and highlight the need for prospective validation through longitudinal protein measurements, immune cell phenotyping, and pathway-level analyses.

For clinical translation of the high-performance predictive models identified in this study, peripheral blood samples should be collected prior to and six weeks following the initiation of ICI therapy. Titers of the CAABs selected by each model are then quantified, fold changes calculated, and incorporated into the model to generate a predictive score. Patients with scores exceeding the model-specific optimal threshold would be classified as at risk for adverse events or as potential responders. Future studies aimed at optimizing blood sampling intervals may further improve the timeliness and accuracy of these predictions.

This study has several limitations. The initial screening phase was restricted to five patients due to practical and financial constraints, which inevitably narrows the scope of this analysis. Identifying approximately 60 CAABs from a proteome-wide panel encompassing over 20,000 potential targets posed a substantial methodological challenge. Given the limited sample size, the presence of variability and possibility of overlooking relevant antigens cannot be excluded. As a result, the present findings provide a partial snapshot of the broader treatment-induced alterations in the antibody repertoire. The specificity of the autoantibody detection platform and selection strategy precluded the use of suitable publicly available datasets, preventing evaluation of model performance across independent or multi-ethnic populations. To achieve a more comprehensive understanding of CAAB dynamics in the context of ICI therapy, future investigations should involve larger and more diverse patient populations and employ unbiased, high-throughput proteomic profiling strategies.

## Data Availability

The raw data supporting the conclusions of this article will be made available by the authors, without undue reservation.
