# Supplementary material for: Machine learning–based insights into circulating autoantibody dynamics and treatment outcomes in patients with NSCLC receiving immune checkpoint inhibitors
Source: Front Immunol. 2025 Oct 3;16:1666030. doi: 10.3389/fimmu.2025.1666030 (PMC12531255; doi:10.3389/fimmu.2025.1666030)
Supplement: Supplementary file 1 [file Table1.docx]

Supplementary Material

# Table S1. Circulating autoantibodies selected in the primary screening (data sheet of Excel)

| **No.** | **Symbol** | **Gene ID** | **Function** | **Location** | **P-value^a^** | **Fold change^b^** | **Selection criteria^c^** |
| --- | --- | --- | --- | --- | --- | --- | --- |
| **1** | SSBP4 | KP01M20 | single-stranded DNA binding | Nucleus | 0.000 | 0.563 | A,B |
| **2** | BACH2 | KP06M23 | Transcriptional regulator | Cytoplasm / Nucleus | 0.001 | 0.527 | A,B |
| **3** | UBTF | KP02N15 | Upstream Binding Transcription Facto | Nucleus | 0.003 | 0.543 | A,B |
| **4** | WDR24 | KP10K20 | mTOR | Lysosome membrane | 0.004 | 1.830 | A,B |
| **5** | FGFR3 | KP07G21 | FGF | Cell membrane | 0.004 | 0.592 | A,B |
| **6** | ITGBL1 | KP02H13 | integrin-related protein | Secreted | 0.005 | 0.603 | A,B,C |
| **7** | HTATSF1 | KP02J21 | transcription | Nucleus | 0.006 | 0.455 | A,B |
| **8** | KLHL18 | KP02G21 | ubiquitination | Nucleus | 0.009 | 0.601 | A,B |
| **9** | NKIRAS1 | KP02I22 | NF-kappa-B | Cytoplasm | 0.010 | 0.376 | A,B |
| **10** | PRDX3 | KP05K11 | Thiol-specific peroxidase | Mitochondria | 0.016 | 1.498 | A,B |
| **11** | BCL2L1 | KP09M17 | apoptosis | Mitochondria | 0.016 | 1.600 | A,B |
| **12** | GATAD2B | KP04L21 | transcriptional repressor. | Nucleus | 0.016 | 1.618 | A,B |
| **13** | ZNF551 | KP01N14 | transcriptional regulation | Nucleus | 0.017 | 0.636 | A,B |
| **14** | STAT1 | KP04K18 | STAT | Cytoplasm / Nucleus | 0.017 | 2.220 | A,B,C |
| **15** | IL4 | KP04O11 | immune | Secreted | 0.020 | 1.453 | A,B,C |
| **16** | PKDCC | KP06N18 | Secreted tyrosine-protein kinase | Secreted | 0.020 | 0.517 | A,B |
| **17** | SOX1 | KP02H22 | Transcription Factor | Nucleus | 0.021 | 0.430 | A,B |
| **18** | DNTT | KP10N15 | DNA polymerase | Nucleus | 0.021 | 1.558 | A,B |
| **19** | PIP4K2A | KP07K16 | second messengers | Cell membrane / Nucleus / Lysosome / Cytoplasm | 0.022 | 0.499 | A,B |
| **20** | DBX1 | KP03E16 | sequence-specific DNA binding | Nucleus | 0.023 | 1.599 | A,B |
| **21** | KRT8 | KP10G18 | keratin | Extracellular | 0.027 | 2.149 | A,B |
| **22** | CSF2 | KP03F15 | immune (GMSCF) | Secreted | 0.027 | 1.710 | A,B,C |
| **23** | PARK2 | KP01H22 | ubiquitin ligase complex | Cytoplasm | 0.028 | 1.625 | A,B |
| **24** | SPATC1L | KP02I23 | Spermatogenesis | Cytosol | 0.028 | 0.449 | A,B |
| **25** | CASP10 | KP02N17 | Caspase | Cytoplasm | 0.030 | 0.638 | A,B |
| **26** | CPB1 | KP02P18 | Carboxypeptidase | Secreted | 0.031 | 0.657 | A,B |
| **27** | VPS18 | KP09O11 | vesicle transport | Endosome | 0.035 | 1.570 | A,B |
| **28** | HES1 | KP05L18 | transcription factors. | Nucleus | 0.040 | 0.523 | A,B |
| **29** | RNASE2 | KP09N11 | non-secretory ribonuclease | Lysosome | 0.042 | 1.657 | A,B |
| **30** | FBXL6 | KP03M14 | ubiquitination | Nucleoplasm | 0.043 | 0.439 | A,B |
| **31** | F13B | KP02I15 | coagulation factor XIII B subunit | Secreted | 0.044 | 0.675 | A,B |
| **32** | STAT4 | KP03E10 | STAT | Cytoplasm / Nucleus | 0.044 | 1.420 | A,B,C |
| **33** | CD40 | KP03J07 | immune | Cell membrane | 0.044 | 1.396 | A,C |
| **34** | ROPN1 | KP02K13 | spermatazoa, cancer-testis antigen | Cell projection | 0.044 | 0.624 | A,B |
| **35** | SCGN | KP09N14 | calcium-binding protein | Cytoplasm / Secreted | 0.045 | 0.417 | A,B |
| **36** | PFKFB4 | KP07K08 | kinase/phosphatases | Nucleoli | 0.046 | 1.745 | A,B |
| **37** | SDC1 | KP08L17 | transmembrane (type I) heparan sulfate proteoglycan | Membrane / Secreted | 0.047 | 1.723 | A,B,C |
| **38** | ECSCR | KP07J15 | angiogenesis | Cell membrane | 0.049 | 1.670 | A,B |
| **39** | ITGAE | KP11F21 | Integrins | Cell membrane | 0.058 | 1.482 | B,C |
| **40** | IL17A | KP03P09 | immune | Secreted | 0.064 | 0.661 | B,C |
| **41** | BIRC3 | KP04I23 | apoptosis | Cytoplasm / Nucleus | 0.068 | 1.512 | B |
| **42** | CCDC3 | KP02O17 | Negatively regulates TNF-alpha-induced pro-inflammatory response | Secreted | 0.070 | 0.682 | B,C |
| **43** | PARK7 | KP05G17 | peptidase | Cell membrane | 0.071 | 2.076 | B |
| **44** | WNT5A | KP04E15 | WNT gene family | Secreted | 0.079 | 1.526 | B |
| **45** | BASP1 | KP10J19 | membrane bound protein | Cell membrane | 0.082 | 2.437 | B |
| **46** | FURIN | KP08N18 | proteases | Cell membrane | 0.083 | 1.701 | B |
| **47** | BCL10 | KP05G11 | apoptosis | Cytoplasm | 0.085 | 0.655 | B |
| **48** | IRF4 | KP05N19 | Interferon Regulatory Factor 4 | Nucleus | 0.087 | 1.523 | B.C |
| **49** | TLR4 | KP06A24 | immune | Cell membrane | 0.098 | 1.408 | B,C |
| **50** | S100A7A | KP10D18 | epidermal differentiation and inflammation | Cytoplasm | 0.120 | 1.839 | B,C |
| **51** | SMAD3 | KP04L10 | intracellular signal transducer proteins | Cytoplasm | 0.125 | 0.655 | B,C |
| **52** | LAMA4 | KP11J06 | Laminin Subunit | Secreted | 0.126 | 1.944 | B |
| **53** | STK11IP | KP10J15 | Serine/Threonine Kinase 11 Interacting Protein | Cytoplasm | 0.140 | 1.806 | B |
| **54** | SNCA | KP09M13 | phospholipase D2 | Cytoplasm | 0.153 | 2.429 | B |
| **55** | IL7 | NP_000871.1 | immune | Secreted | 0.175 | 1.268 | C |
| **56** | CD200 | NP_005935.4 | immunosuppression and regulation of anti-tumor activity | Cell membrane | 0.177 | 3.579 | B,C |
| **57** | MET | KP11N18 | HGF ligand | Cell membrane | 0.178 | 1.409 | B |
| **58** | CXCL2 | NP_002080.1 | immune | Secreted | 0.190 | 1.305 | C |
| **59** | CXCL1 | NP_001502.1 | immune | Secreted | 0.202 | 1.292 | C |
| ^a^ P-values were calculated using Student’s t-test on fold changes from five patients in the pilot study | | | | | | | |
| ^b^ Fold change represents the average value across the five patients | | | | | | | |
| ^c^ Selection criteria: A. Statistically significant (*p* < 0.05); B. Pronounced fold change between before and after treatment (< 0.7 or > 1.4); C. Established immunological relevance | | | | | | | |
